# Under the stethoscope: Unveiling the surge for suicides and mental health concerns among medical professionals in India - A comprehensive literature review

**DOI:** 10.6026/973206300213931

**Published:** 2025-10-31

**Authors:** Rajvi D Chaudhary, Arpit Madhusudanbhai Jani, Shah Karan Kiritkumar, Meghna D Chaudhary, Dipesh G Patel, Nehapriya Prakash

**Affiliations:** 1 Department of General Medicine, Nootan Medical College & Research Centre, Sankalchalchand Patel University, Visnagar, Gujarat, India; 2 Department of Psychiatry, Nootan Medical College & Research Centre, Sankalchalchand Patel University, Visnagar, Gujarat, India; 3 Environmental Health Safety Manager, Hologic, USA; 4 Department of Forensic Medicine, Nootan Medical College & ResearchCentre, Sankalchalchand Patel University, Visnagar, Gujarat, India; 5 Department of General Medicine, Neha Prakash Hospital, Yelahanka, Bangalore, Karnataka, India

**Keywords:** Burnout, medical professionals, mental health, stigma, suicide

## Abstract

Medical professionals in India are experiencing a sharp increase in burnout, depression, anxiety and suicide, especially among students, residents and physicians. Key contributors include academic pressure, long hours, poor support systems and mental health stigma. Anesthesiologists and female doctors are particularly vulnerable, with the COVID-19 pandemic worsening the crisis. Despite growing awareness, evidence-based interventions and safe disclosure mechanisms remain limited. Strengthening nationwide screening, a counseling and institutional mental health policy is urgently needed.

## Background:

The recurrent occurrence of suicides among medical professionals, including students, residents and physicians, has garnered increased attention in the headlines. The demanding and protract nature of medical training, spanning from medical school through residency and beyond, subjects individuals to persistent pressures. Significantly, medical professionals (MPs) exhibit a suicide rate two-and-a-half times higher than the general population [1]. Furthermore, India surpasses the global average for suicide death rates among MPs [2]. There is a critical need for more comprehensive studies in India to elucidate the prevalence and underlying causes of mental health issues, particularly those leading to suicides. It is incumbent upon systems, organizations, medical professionals and all stakeholders to actively engage in thorough investigations and the implementation of interventions aimed at preventing and addressing mental health challenges. The situation's urgency demands immediate action to avert further loss of lives. Therefore, it is of interest to review the surge in suicides and mental health concerns among medical professionals in India.

## Data sources and study selection:

A comprehensive systematic search was conducted on PubMed, Google Scholar and HOLLIS to identify studies on suicide rates, suicidal ideation among medical professionals, mental health issues among medical professionals, barriers to seeking help for mental health issues, suicides among anesthesiologists, mental health issues among anesthesiologists, interventions for suicide prevention and treatment for mental health issues among MPs in India. The timeframe for this search spanned from 2009 to 2023.

## Suicide rates and causes:

Between 2010 and 2019, India documented 358 cases of suicide, encompassing 125 medical students, 105 residents and 128 doctors [3]. Among these instances, males exhibited a higher suicide rate among both medical students and practicing physicians, while female suicides were more prominent among resident physicians. Notably, there were thirty reported suicides between March 2016 and March 2019 [1]. Academic stress, harassment and mental illness account for the majority of suicides among MPs. The primary factors contributing to suicides included longer work hours, inadequate sleep and diet, access to fatal medications and dosages, higher stress levels and unrealistic personal expectations [4]. Academic stress was the most frequently mentioned factor in the 196 medical student suicides reported between 2009 and 2018 [5]. The suicide rate among females was higher than that of males and the most common method was hanging. Even though India's total number of medical seats doubled in ten years, the number of reported suicides increased fourfold, from nine to thirty-seven, in the same time frame [5].

## Mental health issues and burnout among medical students:

"Burnout is a syndrome conceptualized as resulting from chronic workplace stress that has not been successfully managed. It is characterized by three dimensions: (1) feelings of energy depletion or exhaustion; (2) increased mental distance from one's job, or feelings of negativism or cynicism related to one's job; and (3) a sense of ineffectiveness and lack of accomplishment" [6]. Medical students constitute a significant portion of incoming doctors in India. Most students face a high-pressure environment for the first time during their medical school years and are at risk of developing mental health issues because of exam pressure, worry and uncertainty about postgraduate admissions. A study at a tertiary medical college found that most undergraduate medical students reported moderate to high stress levels [7]. Long working hours, lack of sleep, poor nutrition and heavy patient load contribute to burnout and suicide among medical students. A large-scale survey of medical students in India found that 80% of 597 participants experienced burnout [8]. These data suggest that academic stress, burnout and other epidemiological and socioeconomic factors contribute to mental illness. In risk-taking and self-harm inventories, male students scored higher than females [7]. However, there is a lack of research on risk-taking and self-harm behaviors due to stress among MPs in India. Researchers at North Bengal Medical College, Darjeeling, found that many medical students experienced stress, anxiety and depression, with dormitory living and English-based curricula increasing stress [9]. Contrary to Chahal *et al.* [3], females were more impacted than males and tobacco use was linked to stress, anxiety and depression [7]. Substance use disorders and risk-taking behavior among MPs because of stress and mental illnesses need to be evaluated through dedicated research studies. A cross-sectional study at a rural medical institution in Northern India assessed the prevalence of mental health issues among female undergraduate medical students [10]. Out of 367 participants, 40.7% were highly stressed, 36.5% reported high anxiety and 17.9% had moderate to high levels of depression. Most reported sleep issues and irregular exercise patterns. Interpersonal conflicts and family history of psychiatric illness were significantly associated with higher perceived stress [10]. A cross-sectional study of private medical college students in South India reported a high prevalence of depression, anxiety and stress among medical and paramedical students [11]. The final year of medical school was independently associated with higher stress levels. Exam-related pressure remains a key factor impacting MPs' mental health. In a tertiary care setting in Tamil Nadu, medical students showed poor sleep quality and consistent stress throughout their undergraduate training [12], suggesting a link to long-term mental and physical illnesses. Study breaks for students with mental health issues, when necessary, can help them cope with stress and burnout. In a cross-sectional survey, Ameer *et al.* [13] reported that 14% of medical students contemplated self-harm or suicide and 15% engaged in violent behavior. Alcohol and tobacco use were also prevalent. These findings imply that mental health conditions can be fatal for medical professionals and directly impact patient care. During the COVID-19 pandemic, Manglesh *et al.* [14] conducted a cross-sectional survey among Indian medical students and found that 60.3% had mental health problems, with women being more affected than men. Psychological distress was linked to alcoholism, smoking, professional dissatisfaction and uncertainty caused by the pandemic [14]. These findings underscore the need for early detection, counseling and continuous support to address the long-standing mental health burden among medical professionals.

## Mental health issues and burnout among resident and practicing physicians:

Among medical students and practicing physicians, resident physicians have a comparatively higher workload, lack of sleep and concern about exams held during their last year of residency. While searching for the literature, I found very few studies discussing mental health-related concerns among residents and practicing physicians when compared to medical students. Many resident physicians working in COVID-19 facilities of a medical institute in India experienced a significant amount of mental distress [15]. Burnout, depression, anxiety, insomnia and stress were the main symptoms. Anxiety and depression were significantly predicted by burnout and compromised sleep quality [16]. Presence of these factors also negatively affects patient care and relationships between colleagues, senior and junior residents, faculty and residents [16]. A survey revealed that 48.8% of resident physicians of an institute work for 41-60 hours a week and 39.24% showed total burnout with higher stress in first-year residents compared to second and third-year residents [17]. These inconsistencies warrant more studies assessing burnout among different levels of MPs that can help investigators bring possible solutions. In a large-scale survey of registered practicing physicians, significant levels of depersonalization, emotional exhaustion and low levels of personal accomplishments and medical practice were found, indicating high levels of burnout [18, 17].

## Suicide among anesthesiologists necessitates careful consideration:

Anesthesiology residents have better knowledge and easy access to medications that can end life without pain. This can also be the reason, along with other stressors, that they have higher suicide rates than other branches of medicine. In her literature review, Gurunathan [19] pointed out that there is less clear data suggesting higher rates of suicide among anesthesiologists. Still, some older studies across the globe have found that anesthesiologists have a higher risk of suicide by lethal drug administration. They also reported that Anesthetists tend to abuse drugs due to knowledge and easy access to drugs and stress related to the job. In their review article, Verma and colleagues [20] pointed out that anesthesiologists are held responsible for any unfortunate accident rather than surgeons and surgery itself, which predisposes them to higher stress levels, decreased job satisfaction and constant worry about the future. As frontline workers and critical care specialists, Anesthesiologists felt higher stress and mental health-related issues during COVID-19 [21]. An online survey of Anesthesiologists in India found that most Anesthesiologists think they have higher stress levels than other branches of medicine [22]. More studies are needed to assess the reasons behind stress, mental health issues and suicides among anesthesiologists. Self-administration of lethal drugs for suicide signals higher authorities to take further steps to prevent these unfortunate incidents.

## Stigma on seeking help for mental health issues:

The stigma around mental health is one of the major factors that hold back MPs from asking for help when needed. One of the reasons could be due to the negative effect on MPs' careers because of disclosure of their mental illness and, ultimately, questions on patient care. Not seeking help when needed can lead to worse outcomes in the long term. A study conducted at a government medical college in Puducherry found that compared to physical health-related concerns, most medical students reported stigma, confidentiality problems and a lack of awareness when it came to getting help for mental health issues [11]. It was found that suicidal thoughts were present in 53.6% of medical students at a medical college in Delhi [8]. Despite widespread awareness of the definition of suicide and the harm it can do to a person and their family, 4.9% of students reported having seriously considered suicide and 2.6% said they had attempted suicide at least once in their lifetime. Being a healthcare professional did not eliminate mental health stigma.

## Mitigating burnout, mental health issues and suicides - prevention and intervention strategies:

Echoing Desiderius Erasmus's wisdom that "Prevention is better than cure," averting the onset of illnesses transcends the challenges of addressing them post-manifestation. The prevention of mental health issues, though conceptually simpler than its implementation, stands as an imperative to safeguard invaluable lives. The root causes of burnout lie within healthcare organizations and systems, including overwhelming workloads, inefficient processes, administrative encumbrances, work-home conflicts and a deficiency in physician influence or authority [23]. In their publication, Menon *et al.* [24] proposed several strategies to address burnout among physicians, including establishing work schedules that support work-life balance, leveraging automation for specific documentation tasks to enhance efficiency, prioritizing physician wellness and fostering the development of physician leaders capable of instigating positive changes. Sanjukta and their associates [2] explore the evaluation of suicide risk in medical students. This involves assessing their history for previous psychiatric diagnoses, suicide attempts, identifiable stressors and precipitants. Educating students on recognizing warning signs is crucial for suicide prevention and raising awareness about suicidal ideation. Reciprocal causation exists between burnout and mental health issues, each exacerbating the other's impact. The focus should be on discerning burnout syndrome's manifestations and devising multifaceted strategies to address them [25, 17]. Comprehensive nationwide studies and exploring additional correlates will be imperative for comprehending this phenomenon and formulating preventative and management measures [26]. Swift and decisive actions are warranted to redress this pressing issue [27]. The General Medical Council of the United Kingdom [GMC UK] encourages students struggling with mental health issues to take breaks from their studies [28]. Taking breaks during challenging moments can support MPs in managing stress more effectively. It offers them time and a conducive environment for self-reflection and, if necessary, seeking treatment. Healthcare institutes in India need separate entities for mental health screening policies, counseling and treatment for MPs to provide confidential and easy-to-reach-out mental health services [24, 25]. Sanju and Nitin addressed the concept of Occupational Mental Health Services [OMHS] in their paper [29]. The concept of OMHS includes providing mental health services to medical students, doctors and allied healthcare professionals through commission or by building the institutions own OMHS. Along with OMHS, introducing technological methodologies and student wellness centers may increase the reach of preventative measures that can prevent suicidal attempts and at-risk behaviors [2]. Comprehensive solutions involve addressing these drivers through organizational changes, such as locally tailored practice modifications, increased clinical support and individual interventions like mindfulness-based stress reduction and community-focused programs [23]. The concerted collaboration between healthcare systems and individual physicians is paramount in successfully addressing burnout, mental health challenges and mitigating the risk of suicides within the medical profession.

## Gaps in literature considering suicides, mental health and interventions:

While the General Medical Council (GMC) in the United Kingdom has developed structured guidelines and institutional support systems for medical professionals grappling with mental health challenges [17], India continues to face a significant gap in this area-particularly concerning protocols to protect the careers of medical students who disclose mental illnesses. Proactive initiatives are urgently needed to address these deficiencies, ensuring that mental health disclosure does not jeopardize professional advancement. Elevated levels of burnout are consistently reported among female clinicians compared to males, with practitioners in surgical specialties demonstrating a higher prevalence of burnout than those in medical branches [28]. However, further research is required to validate these findings across diverse regions and healthcare settings in India. In their study, Chakraborty *et al.* highlighted alarming rates of anxiety, stress and depression among medical students, emphasizing the urgent need for counseling services and institutional support. They also underscored the importance of additional research to identify further contributing factors [9]. Moreover, limitations such as short follow-up durations, narrow study scopes and insufficient use of randomized controlled trial designs hinder the generalizability of intervention-based research on burnout. The reliability of findings across demographic variations and practice settings remains uncertain. West *et al.* stressed that greater methodological rigor and uniformity are essential in developing new metrics for assessing burnout and its determinants [23]. Few Indian studies have comprehensively examined the complex interplay between suicide rates, mental health challenges and systemic stressors among medical professionals. This scarcity of research not only highlights a critical gap in understanding but also underscores the need for evidence-based, culturally contextualized interventions and sustained institutional reforms to protect the mental well-being of healthcare workers.

## Discussion:

Across medical institutions in India, numerous studies have revealed distressing levels of burnout, chronic stress, reluctance to seek psychological help and even suicidal ideation among medical professionals [30, 31]. The reluctance of MPs to seek help is often reinforced by pervasive stigma, fear of professional repercussions and societal misconceptions about mental illness. These barriers perpetuate silence and delay necessary interventions, aggravating the mental health burden within the medical community. Additionally, insufficient awareness regarding available mental health resources further limits access to care. This underscores the necessity of comprehensive institutional strategies-such as early screening programs, preventive counseling and confidential mental health services-to foster timely intervention. Establishing a psychologically supportive and stigma-free environment within medical colleges and hospitals is imperative for sustainable change. A recent study conducted in a peripheral medical college in West Bengal demonstrated the adverse psychological impact of the COVID-19 pandemic on medical students [32], reflecting heightened stress, anxiety and emotional exhaustion during this period. However, the broader trajectory of mental health issues among medical professionals-both preceding and following the pandemic-remains insufficiently explored in Indian literature. This paucity of longitudinal data underscores the urgent need for multicentric, large-scale studies aimed at identifying risk factors, preventive strategies and institutional determinants influencing mental health outcomes among India's medical workforce.

## Conclusion:

The growing mental health crisis among medical professionals demands immediate recognition and action. Fostering a culture of empathy, open communication and psychological safety within the healthcare system is essential. By prioritizing mental well-being, strengthening support networks and promoting timely interventions, we can safeguard the lives and resilience of those who dedicate their own to healing others.
